# Carbon-Coated, Diatomite-Derived Nanosilicon as a High Rate Capable Li-ion Battery Anode

**DOI:** 10.1038/srep33050

**Published:** 2016-10-07

**Authors:** Brennan Campbell, Robert Ionescu, Maxwell Tolchin, Kazi Ahmed, Zachary Favors, Krassimir N. Bozhilov, Cengiz S. Ozkan, Mihrimah Ozkan

**Affiliations:** 1 Materials Science and Engineering Program, Department of Mechanical Engineering, University of California, Riverside, Riverside, CA 92521 (USA); 2 Department of Chemistry, Department of Electrical and Computer Engineering, University of California, Riverside, Riverside, CA 92521 (USA)

## Abstract

Silicon is produced in a variety of ways as an ultra-high capacity lithium-ion battery (LIB) anode material. The traditional carbothermic reduction process required is expensive and energy-intensive; in this work, we use an efficient magnesiothermic reduction to convert the silica-based frustules within diatomaceous earth (diatomite, DE) to nanosilicon (nanoSi) for use as LIB anodes. Polyacrylic acid (PAA) was used as a binder for the DE-based nanoSi anodes for the first time, being attributed for the high silicon utilization under high current densities (up to 4C). The resulting nanoSi exhibited a high BET specific surface area of 162.6 cm^2^ g^−1^, compared to a value of 7.3 cm^2^ g^−1^ for the original DE. DE contains SiO_2_ architectures that make ideal bio-derived templates for nanoscaled silicon. The DE-based nanoSi anodes exhibit good cyclability, with a specific discharge capacity of 1102.1 mAh g^−1^ after 50 cycles at a C-rate of C/5 (0.7 A g_Si_^−1^) and high areal loading (2 mg cm^−2^). This work also demonstrates the fist rate capability testing for a DE-based Si anode; C-rates of C/30 - 4C were tested. At 4C (14.3 A g_Si_^−1^), the anode maintained a specific capacity of 654.3 mAh g^−1^ – nearly 2x higher than graphite’s theoretical value (372 mAh g^−1^).

Magnesiothermic reduction can be used in conjunction with a low-cost and abundant SiO_2_ source to produce nanoSi-based Li-ion battery anodes significantly more efficiently. Diatomaceous earth is a friable sedimentary rock (diatomite, DE) which has been deposited over millions of years in aquatic environments, leaving massive deposits of thousands of square acres and thousands of feet deep^1^. It is composed of the deceased, fossilized frustules of diatoms. Diatoms are a diverse group of photosynthetic, single-celled microorganisms that exist as an algae that are responsible for a significant contribution of oxygen (O_2_) production in Earth’s atmosphere. Their frustules are principally biomineralized as SiO_2_, containing amorphous quartz and opaline phases with small amounts of Fe_2_O_3_ and Al_2_O_3_ impurities^1^,^2^,^3^. The SiO_2_ purity can range in diatomaceous earth, depending on the source, from 75.68–96.02 wt.%^1^.There are massive diatomite deposits around the world; the United States is the world’s greatest producer of natural diatomite, having produced 770 metric tons of diatomite in 2013^4^. Diatomite, therefore, represents an abundant source of high purity SiO_2_, and serves as an important potential precursor to silicon (Si).

In order to convert SiO_2_ structures and phases into nanoSi made up of extremely small particles (down to nearly 5 nm), a heat scavenging agent may be used in conjunction with magnesiothermic reduction. An excellent heat scavenger is common table salt (NaCl), as seen in the work of Favors, *et al*.^5^,^6^,^7^, which has a positive enthalpy of fusion and absorbs a significant amount of the heat produced from the highly exothermic Mg reduction (ΔH = −586.7 kJ mol_SiO2_^−1^)^7^. Ultimately, the thermodynamic offset from the endothermic NaCl phase change to liquid prevents local melting of the Si during the reaction. In this work, nanoSi particles were observed after converting purified diatoms frustules to nanoSi using magnesiothermic reduction. In addition, the morphologies of the diatoms frustules were conserved after being converted into elemental Si from the reduction reaction, resulting in nanoSi particle-composed diatom frustule structures. These naturally occurring structures are advantageous for use in Si anodes for lithium-ion (Li-ion) batteries because of the macroporous nature of the frustule structures, which allows easy access for the electrolyte, and the nanoscaled particle size of the nanoSi, which resists pulverization during expansion/contraction upon lithiation/delithiation^8^,^9^,^10^. The straightforward synthesis of C-coated frustule-like nanoSi from DE is depicted in the schematic in Fig. 1. Other naturally occurring structures benefit electrode performance, such as in pyrolytic biomass-based electrodes^11^. It is also possible to couple a Si-based electrode with other high-capacity next-generation cathode materials, such as sulfur, which has a theoretical capacity of 1675 mAh g^−1^, nearly 10x that of lithium cobalt oxide^12^. The highly mesoporous nature of the resulting frustule-like nanoSi, coupled with its macroporous frustule-like architectures, provide an ideal bimodality that can help the structure cope with a massive theoretical volume expansion of 280% upon Li alloying (it should also be noted that experimental evidence shows the expansion could be much greater, upwards of 490%)^13^.

## Results

**Figure 2:**
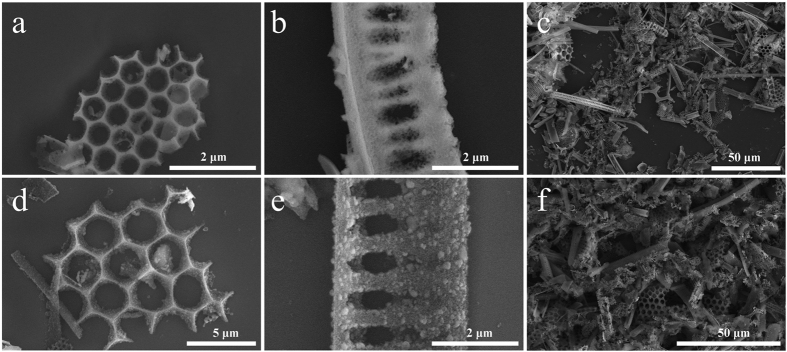
SEM characterization of 2 distinct types of DE frustule fragments with unique geometries (**a,b**), lower-magnification SEM of the powder made up of DE (**c**), the corresponding geometries of nanoSi structures derived from DE frustules (**d,e**), and lower magnification SEM of the powder made up of DE-derived nanoSi (**f**).

Scanning electron microscopy (SEM) was used to study the structure of the DE as-obtained, as well as after the magnesiothermic reduction. In Fig. 2a–c, the morphology and scale of the DE microstructures can be observed. Since DE is composed of an array of diatoms frustules with diverse morphologies, individual frustules with typical structures are noticed in Fig. 2a,b, with a hexagonal honeycomb architecture and an elongated fence-like architecture, respectively. A lower magnification SEM micrograph captures the true diversity of the frustule structures in Fig. 2c. The features in the frustules vary in shape, size and dimension, and exist in both the nano and microscale. For example, the hexagons of the frustule in Fig. 2a average ca. 530 nm, the spacing between the pillars of the frustule in Fig. 2b vary from ca. 180 nm to >400 nm, and the pillars themselves are approximately 200 nm in diameter. The overall size of the frustule fragments, however, tend to be on the order of several microns. In addition to the as-obtained DE, the DE-derived post-reduction nanoSi structures were studied using SEM (Fig. 2d–f). The structures in Fig. 2d–f have also been C-coated via CVD. Post-reduction, the morphologies of the DE frustule fragments are well maintained. Fig. 2d shows a hexagonal honeycomb architecture similar to the DE frustule from Fig. 2a. The macroporosity (exhibiting voids >50 nm) of the hexagons is preserved along with the structure and morphology of the overall frustule fragment. The same phenomenon is also observed with the elongated fence-like architecture of the DE frustule in Fig. 2b, being largely preserved after the reduction as shown in Fig. 2e. The diverse array of DE frustule fragments are nearly indistinguishable from the post-reduction frustule-templated nanoSi structures on the microstructure level, as can be seen from Fig. 2f. However, the surface morphology of the frustule-shaped nanoSi structures is noticeably different. It is apparent from Fig. 2d,e that upon reduction of SiO_2_ to Si, the resulting microstructures are ultimately composed of smaller Si particles, giving a rough, spongy appearance.

**Figure 3:**
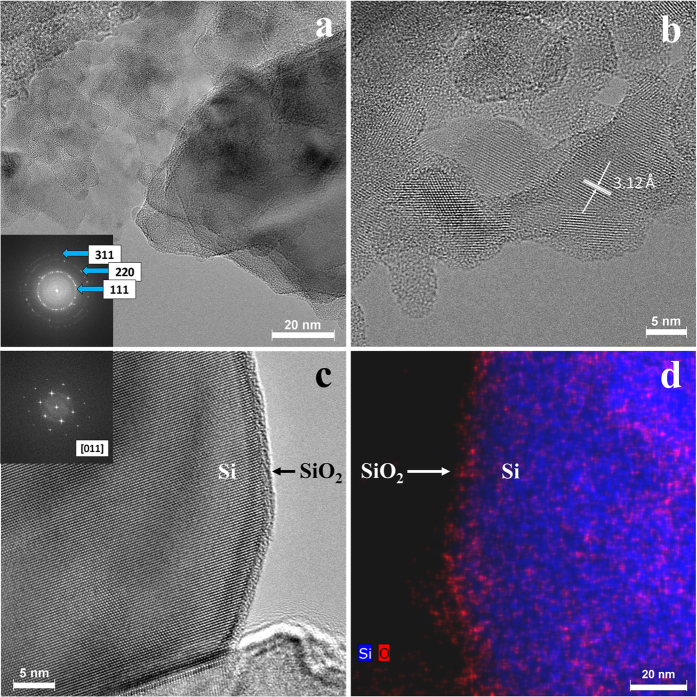
HRTEM analysis of bare DE-derived nanoSi, including Si crystals of various orientations and the indexed selected area electron diffraction pattern as an inset (**a**), select nanoSi particles showing the d-spacing of crystalline Si (**b**), a select larger Si particle with well-distinguished Si core and amorphous surface layer with FFT inset (**c**) and a similar larger particle analyzed by dark-field EDX mapping showing the Si core and oxide surface layer (**d**).

Analysis of the nanostructure of the DE-derived nanoSi was carried out using high-resolution transmission electron microscopy (HRTEM). Figure 3 gives details of the bare nanoSi, before the CVD C-coating step. Multiple crystals are observed in Fig. 3a, for which a selected area electron diffraction pattern (shown as inset in Fig. 3a) was also obtained. The ring SAED pattern is consistent with cubic Si structure with rings corresponding to the 111, 220, and 311 reflections, respectively. The higher-magnification HRTEM micrograph in Fig. 3b captures the size of smaller Si particles. Additionally, it can be seen that the crystalline Si (identified by lattice fringes) are encapsulated by a native amorphous oxide layer on the surface (no lattice fringes). In this image, the d-spacing was also measured to be 3.12 Å, the characteristic spacing for Si. Fig. 3c very clearly shows a larger Si particle with a well-defined amorphous oxide surface layer (FFT shows crystal orientation=[011] for the underlying Si substrate within the particle). This structure is confirmed with a similar particle in Fig. 3d, showing elemental mapping by combined energy-dispersive X-ray spectroscopy (EDX) and high angle annular dark field (HAADF) STEM imaging. With Si highlighted in blue and O in red, a strong elemental distinction can be made between the bulk particle’s Si and the SiO_2_ making up the surface layer. For the structural stabilization of the Si anode over the first several cycles, and establishing an optimal SEI layer, this oxide layer is critical^14^.

**Figure 4:**
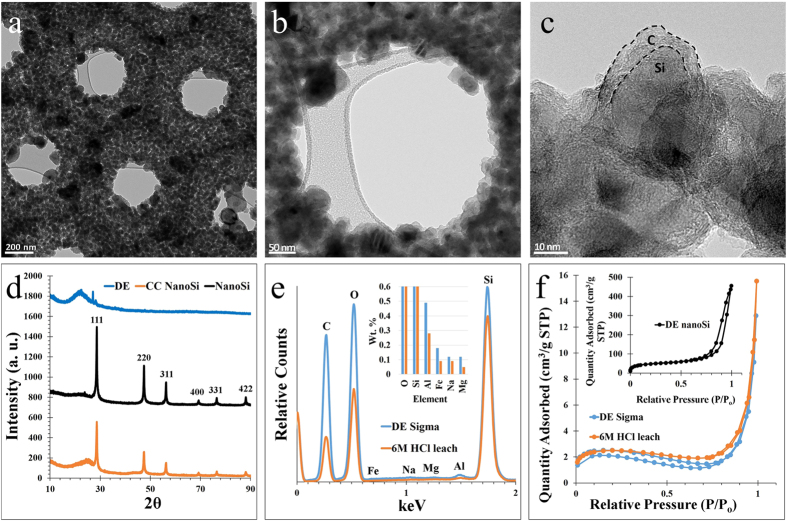
Low to high magnification TEM characterization of a hexagonal honeycomb-shaped frustule-like nanoSi structure showing the SiNPs (**a–c**), XRD spectra of DE, nanoSi, and C-coated nanoSi (**d**), the EDX spectrum and elemental composition of DE before and after HCl leaching (**e**), and BET N_2_ adsorption isotherms of DE before and after purification, and nanoSi (**f**).

Additional TEM characterization was conducted to observe the final nanostructure preservation of the nanoSi after C-coating. Preservation of the overall frustule-like architecture of the nanoSi structures is further confirmed in Fig. 4a, where a hexagonal honeycomb arrangement is observed. At this magnification, various pore sizes can also be observed. Macropores (>50 nm) are provided by the inherent honeycomb architecture of the nanoSi structures, and visible mesopores are generated as a result of the magnesiothermic reduction. Fig. 4b shows a higher magnification micrograph capturing the inner diameter of a hexagon present in the frustule-like nanoSi structure. In this image, the size of the post-reduction silicon nanoparticles (SiNPs) can be observed, ca. 10 s of nanometers. The SiNPs that make up the frustule-like nanoSi structures are well below the critical dimension D_c_ of ~150 nm, below which Si nanoparticles have a high enough fracture toughness to be able to endure lithiation repeatedly without fracturing^15^. From Fig. 4c, the carbon nano-coating can be clearly distinguished from the SiNP, with a thickness ca. 5 nm. The C-coating of the nanoSi is clearly amorphous from the lack of lattice fringes in HRTEM, as also from the low-intensity, broad peak in the powder X-ray diffractometry (XRD) spectrum (Fig. 4d).

XRD analysis also shows the transformation from the impure biosilica of the DE into nanoSi, and ultimately the C-coated nanoSi structures (shown in blue, black and orange, respectively, in Fig. 4d). Here, the (101) plane of DE’s quartz phase is identified, as well as a few smaller impurity peaks. After magnesiothermic treatment, the characteristic peaks of nanoSi (corresponding to reflections 111, 220, 311, 400, and 331) are present in both the bare and C-coated nanoSi spectra in Fig. 4d. Along with the characteristic nanoSi peaks, there is a relatively small amorphous peak present around 2θ ~24°, indicating a stabilizing native oxide surface layer on the nanoSi particles^16^,^17^. The C-coated nanoSi spectrum shows the amorphous carbon peak at 2θ ~25, indicating a successful CVD carbon coating. Energy-dispersive X-ray spectroscopy (EDX) allows Fig. 4e to show the elemental distribution within the DE powder, as well as the relative wt. % of elements before and after leaching the powder with 6 M HCl at 75 °C for 12 hours (inset). A significant reduction in unwanted impurities can be seen after the HCl leaching; for example, Al was reduced by roughly 1/3 and Fe was reduced by nearly 1/2 in terms of overall weight. The HCl-leached DE was subsequently used to produce the magnesiothermic reduction product (frustule-like nanoSi) used in the Li-ion battery performance testing. The Brunauer-Emmett-Teller (BET) method was used to obtain pore structure and surface area data on the DE, HCl-leached (purified) DE, and the frustule-like nanoSi (Fig. 4f). The nitrogen (N_2_) adsorption isotherms reveal that the only material to show mesoporous characteristics is the nanoSi (inset). The hysteresis of the DE indicates a more macroporous structure, and the inset N_2_ isotherm demonstrates hysteresis in the mesoporous region upfield of 0.8 P/P_o_, which is attributed to capillary condensation in pores between 2 and 50 nm^18^,^19^. The specific surface area of the as-purchased DE vs. the nanoSi proved to be drastically different; the DE BET surface area was measured at 7.3 cm^2^ g^−1^, while the frustule-like nanoSi powder had a measured BET surface area of 162.6 cm^2^ g^−1^.

## Discussion

Metallurgical Si production is typically an energy-intensive process. The standard process of carbothermic Si production is highly endothermic; it is described by the following reaction:
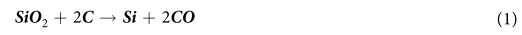


The carbothermic reduction of SiO_2_ shown in Eq. 1 requires 12.5 MWh per ton of Si, and temperatures in excess of 1800 °C^20^,^21^. As an alternative method of Si production that requires far less energy input, magnesiothermic reduction has been employed. The overall reaction is described in Eq. 2.
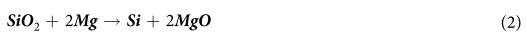


This reaction, in contrast to carbothermic reduction, can be carried out at 650–700 °C^22^,^23^,^24^. Furthermore, use of Mg as the reducing agent in this reaction tends to lead to the production of interconnected SiNPs rather than bulk Si, making it ideal for producing electrode active material on the nanoscale. The combination of the use of magnesiothermic reduction and the use of diatomite as an abundant, nanostructured SiO_2_ feedstock leads to the ability to produce a greener, high-performance Si anode active material for Li-ion batteries. Shen, *et al*. were apparently the first to demonstrate the fabrication of magnesiothermically-reduced DE into an electroactive anode for Li-ion batteries. They report on the cycling of DE-based Si anodes for up to 30 charge-discharge cycles, although the capacity fading is drastic (achieving only 633 mAh g^−1^ after 30 cycles) with a current density of 0.2 mA cm^−2 ^8. Furthermore, Li, *et al*. conducted more thorough characterization of magnesiothermically-reduced DE, showing 10–20 nm Si crystallites with a native oxide layer for cycling stability. They were able to achieve slightly above 750 mAh g^−1^ after 150 cycles, demonstrating good cycle stability. In their synthesis, they introduced NaCl as a heat scavenger which has also been done in other Mg-reduction nanoSi synthesis strategies^25^,^5^,^6^,^7^. The use of such a heat scavenger prevents the highly exothermic reaction from approaching the melting point of Si (1414 °C). In both examples of DE-based nanoSi as Li-ion battery anode materials, C-coating was employed to enhance the conductivity of the nanoSi structures. Regarding polymer binders, previous works have utilized sodium alginate (NaA) and sodium carboxymethyl cellulose (NaCMC). The mechanical properties of organic polymer binders vary widely; in this work, polyacrylic acid (PAA) was used for the first time for DE-based nanoSi in Li-ion batteries. PAA has been shown to increase the efficiency of Si-based anodes, and due to its high concentration of carboxylic groups may function as a superior binder for Si anodes (this is evidenced by lower swellability in the presence of carbonates compared to CMC, greater contact between PAA and SiO_x_/functional groups on the C-coating during lithiation, and Li^+^ hopping through the nanoscaled PAA binder)^26^,^27^,^5^. The enhanced mechanical properties, such as elasticity, are largely derived from an H-bond network formed between H-bond donors and acceptors between the PAA COOH groups and the surface functional groups of the active material (i.e. SiO_x_)^28^. Therefore, in this case, PAA is hypothesized to enhance the rate capability of the DE nanoSi anodes.

**Figure 5:**
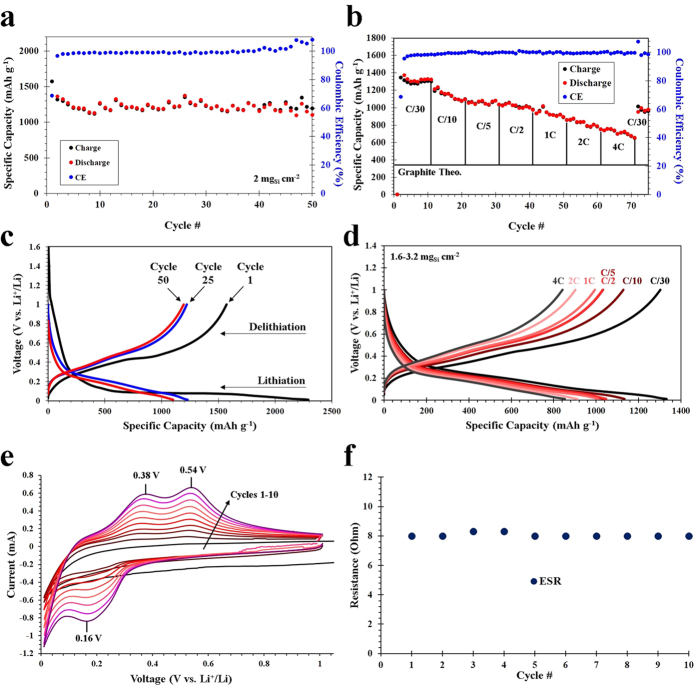
Electrochemical characterization of DE-derived nanoSi-based electrodes, including charge-discharge cycling performance for 50 cycles at C/5 based on Si (**a**), C-rate testing for 75 cycles at C-rates from C/30 – 4C (**b**), voltage profiling of the charge-discharge data at C/5 for cycles 1, 25 and 50 (**c**), voltage profiling of various C-rates (**d**), CV for cycles 1–10 (**e**) and the ESR values for cycles 1–10 based on EIS analysis (**f**).

50 cycles of charge-discharge cyclability testing at a rate of C/5 based on Si (1C = 3.579 Ah g_Si_^−1^), as well as rate capability testing up to rate of 4 C over 75 cycles was performed on the anodes. C/5 was chosen as a benchmark cycling rate for the DE-based nanoSi anodes for 50 cycles, because it is theoretically a 5-hour charge and discharge. The initial cycle was run at a rate of C/50 to establish a stable solid-electrolyte interphase (SEI) on the surface of the Si particles. The cycling results can be seen in Fig. 5a, where the 2^nd^ cycle (1^st^ at C/5) yielded a specific discharge capacity of 1364.8 mAh g^−1^, while the 50^th^ cycle maintained a reversible capacity of 1102.1 mAh g^−1^. A calculation based on these results show that after 50 cycles,>80% capacity retention is preserved. After 50 charge-discharge cycles, the Coulombic efficiency was consistently above 99.9%. The active material areal loading for the C/5 cycling was 2 mg_Si_ cm^−2^. Figure 5b depicts the rate performance of the DE-based nanoSi anodes, which was again cycled once at C/50 to establish the SEI layer, followed by 10 cycles each at C/30, C/10, C/5, C/2, 1C, 2C, 4C, then back to C/30. The capacity retention of each 10-cycle series for the various C-rates (C/30 – 4C) were 67.4%, 90.4%, 98.3%, 95.0%, 88.4%, 87.1% and 88.7%, respectively. The discharge and charge capacities are shown for each cycle, and directly compared to the theoretical specific capacity of graphite (372 mAh g^−1^). After 10 cycles of each rate (C/30 – 4C) cycling, the reversible discharge capacity is 1322.3, 1095.5, 1034.1, 971.5, 892.3, 787.3, and 654.3 mAh g^−1^, respectively. This is extremely good performance for a Si active material, especially considering the relatively high Si areal loading of 1.6–3.2 mg_Si_ cm^−2^ (performance and Si loading was averaged). Other works on Si anodes demonstrate very good rate performance, but with relatively low areal loading (0.3 mg_Si_ cm^−2^, for example)^29^. The capacity fading is further illustrated through the voltage profiling in Fig. 5c,d, where the 1^st^, 25^th^ and 50^th^ cycles are compared (Fig. 5c), and the widening of the voltage plateaus as a function of cycling rate (current density) is apparent (Fig. 5d). The plateaus present in the voltage profiles agree well with the CV measurements in Fig. 5e, with the highly evident peaks associated with alloying (0.16 V) and dealloying (0.38 V, 0.54 V) in the half-cell.

**Figure 6:**
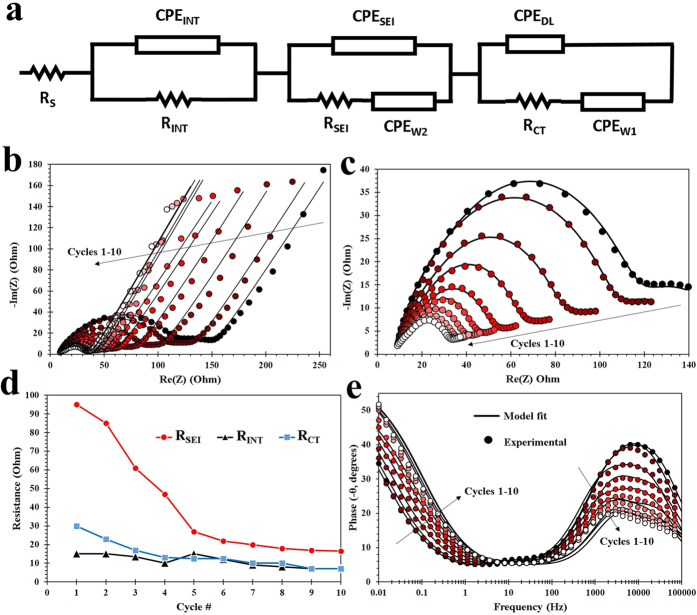
EIS analysis of the DE-derived nanoSi electrodes assembled in a Li-ion half cell, including the EEC based on modeled EIS data (**a**), standard Nyquist plots for 10 cycles including fitted data (**b**), enlarged semi-circle/high-frequency region of the Nyquist plots (**c**), SEI resistance, internal impedance and charge transfer resistance data for 10 cycles (**d**), and Bode plots for 10 cycles including fitted data (**e**).

Extensive EIS analysis was conducted on the DE-derived nanoSi anodes. Figure 6a shows an electrical equivalent circuit (EEC) used for modeling the test electrode by using its electrochemical impedance data. Such data is obtained from electrochemical impedance spectroscopy (EIS), where a small sinusoidal input is overlaid on a DC signal in order to measure linear system response over some frequency range. For the present investigation, we utilized PEIS (potentiostatic EIS) whereby we held the test cell under a fixed voltage while applying a 10 mV sinusoidal input. The resultant impedance data contain critical information about the electrode’s internal electrochemistry. The EEC shown in Fig. 6 consists of individual lumped circuit elements that serve to isolate some of the electrochemical steps that take place during charging or discharging in the Li-ion battery. This is possible because time constants for these steps are usually distinguishable. R_S_ is often expressed as equivalent series resistance (ESR), which largely quantifies electrolyte resistance. Figure 5f shows that this value remains relatively constant throughout the first 10 cycles. Conductivity of carbon additives and metallic extensions within the electrode material can also contribute to this resistance. This step is usually the fastest, and therefore information pertaining to it is found at the highest frequency end of impedance spectrum. Z_INT_, composed of CPE_INT_ and R_INT_, is the impedance due to imperfect contact within the nanostructured Si electrode. This impedance determines the rate at which electrons travel through the active material, specifically. Constant phase elements, or CPEs, are imperfect capacitances arising from non-ideal spatial distribution of chemical and tactile nature of the interfaces involved. These capacitors are charged and discharged according to governing principles, but their effects are not registered in the DC regime. However, quantizing them improves any EEC-based model of a LIB for the purpose of battery management. The second parallel impedance branch characterizes the SEI. A second constant phase element titled CPE_W2_ is also located in this branch. This latter element quantizes the capacitance due to chemical gradient arising from limited diffusion rates of Li ions in the electrolyte near the electrode surface. CPE_W1_, a third constant phase element in the third parallel branch, is used to characterize diffusion of Li^+^ in the solid state (i.e. electrode material, nanoSi). The third parallel branch also quantizes the capacitive nature of the double layer formed at the electrode-electrolyte interface, denoted as CPE_DL_. R_CT_ is the rate-limiting element associated with redox reactions taking place during lithiation and delithiation.

EIS analysis demonstrates self-stabilization of the anode during the first 10 cycles. Evolution of resistive behavior of 3 electrochemical steps within our test cell were characterized via R_INT_, R_SEI_, and R_CT_ (Fig. 6d). R_CT_ decreases as expected during the first 10 cycles. This is due to improving electrochemical conditions for redox reactions over the first few cycles. This stabilizing behavior in R_CT_ gives evidence for good cyclability of the test cell. Evolution of R_INT_ can be characterized into 3 phases by 3 series of cycles: #1–4, 5, then 6–10. The resistance that characterizes the integrity of internal electronic conduction within electrode active material decreases during the first phase (1–4), spikes in the second, then begins to decrease again throughout the third phase. This trend can be explained by a large change in the Si nanostructure at the 5^th^ cycle. This phenomenon at the electrode nanostructure is evidence of the electrode’s tendency to self-stabilize. One explanation for the sudden spike in R_INT_ in the 5^th^ cycle is that majority of electrode wetting by electrolyte was completed by this time, and the resultant increase in “total current” through electrode caused the change in its nanostructure we observe on the 5^th^ cycle. One evidence for this hypothesis is that Q_INT_, which assigns an overall capacitance value to the totality of imperfect contacts within active material nanostructure, decreases comparatively sharply before the 5^th^ cycle. This can be because of increased available surface area for lithiation to occur led to a decreased charge time in the measured capacitance. A drop followed by stabilization in R_SEI_ is essential for any LIB cell with satisfactory lifetime. Trend observed for R_SEI_ over the first 5 cycles also support our theory regarding the completion of our electrode wetting with electrolyte at the 5^th^ cycle. R_SEI_ decreases sharply until the 5^th^ cycle, thereafter decreasing a much slower rate and stabilizing. This agrees with the idea that the SEI formation process (involving irreversible reactions with Li ions and other reactive species present with impedance significantly larger than that of reversible lithiation) over electrode surface stops at the 5^th^ cycle, when electrolyte wetting also finishes. Figure 6e shows a Bode (phase vs. log-frequency) plot from the impedance data obtained. An important point of note here is the remarkable increase in the phase angle (shown as negative of its actual value) with cycles #1–10. This increase suggests that overall impedance of the cell evolved from that of a Warburg element toward a more non-ideal end (similar to a CPE with an n value increasing from 0.5). The electrochemical nature of the cell’s impedance became more distributed spatially at the nanoscale, but its overall impedance (not shown in these figures) decreased by half over the first ten cycles. Figure 6b,c demonstrate the accuracy of the fitting algorithms used. Figure 6c shows the three semicircles corresponding to the three impedance branches highlighted in this analysis. The two higher frequency semicircles are largely overlapping, and the semicircle for double layer impedance is smaller and upfield, predominantly noticeable in cycles 1–2.

## Methods Section

The experiments were carried out as follows: DE was purchased from Sigma Aldrich (Celpure, 98% SiO_2_), and manually milled for 1 hour. DE was then loaded into high-density polyethylene centrifuge tubes, and mixed with 12 M HCl, and held at 75 °C in a thermostated water bath. After approximately 2 days of HCl leaching, the DE was washed thoroughly with ultrapure H_2_O, followed by absolute ethanol (EtOH). The DE was then dried under vacuum at 100 °C, milled, and mixed with NaCl in a weight ratio of 1:10 (DE:NaCl). Ultrapure H_2_O and EtOH was added to this mixture, which was then stirred and slowly evaporated on a hot plate at 80 °C. Once the H_2_O and EtOH was evaporated, the DE-NaCl mixture was milled and mixed into a vial with Mg metal such that the resulting weight ratio was 1:10:0.9 (DE:NaCl:Mg). This mixture was thoroughly vortexed, and then loaded into a stainless steel Swagelok reactor inside an Ar-filled glovebox. The reactor was sealed, then removed and placed into a tube furnace. The quartz tube was purged with Ar gas, and the temperature was ramped at 5 °C per minute to 700 °C and held for 2 hours. The furnace was then cooled back to r.t., and the crude product was removed from the reactor. To remove impurities, the crude product was mixed with 6 M HCl and allowed to react for 24 hours, with occasional stirring. During this time, the purification of nanoSi was apparent from the color change from blackish-brown to light golden brown. Afterwards, the nanoSi was neutralized to pH 7 with several washes with ultrapure H_2_O and EtOH. Once it is neutralized, the nanoSi product was dried under vacuum at 80 °C overnight. The nanoSi powder was C-coated in a separate tube furnace under slight vacuum (600 Torr). First, a mixture of Ar and H_2_ gas was flowed (100 SCCM each). The temperature was ramped at approximately 35 °C per minute to 950 °C and held for 20 minutes. During this 20 minutes, ethylene gas (C_2_H_2_) was flowed at 50 SCCM. After the 20 minutes, the C_2_H_2_ was shut off, and the furnace cooled to r.t. The C-coated nanoSi was mixted with acetylene black (AB) and polyacrylic acid (PAA), using EtOH as the carrier fluid/solvent for slurry-casting the electrodes on copper foil and subsequent electrochemical characterization.

Battery performance testing was conducted in the CR2032 coin cell form factor for all batteries. The cells were fabricated using working electrodes comprised of frustule-like nanoSi active material mixed with AB and PAA, cast onto Cu foil current collectors with EtOH as the carrier fluid. For CV and EIS measurements, the nanoSi:C:PAA mass ratio of the electrodes was 7:2:1, and for the cycling data and C-rate testing, the ratio was 5.5:3.5:1. Microporous polypropylene (PP) was used as the separator (Celgard 2300), and Li metal foil was used as the counter electrode. The electrolyte used was 1 M LiPF_6_ in a 1:1 v/v FEC/DMC solvent system for all batteries. All cells were fabricated inside an Ar-filled glovebox (VAC Omni-lab). For charge-discharge cycling data and C-rate testing, the cells were cycled from 0.01 to 1 V on an Arbin BT2000. The CV and EIS data was collected on a Bio-logic VMP3 tester. For CV, a scan rate of 0.1 mV/s was used, and EIS was performed initially from open circuit voltage (E_OC_) and subsequently scanned from 0.1 Hz to 1 MHz after each CV cycle.

SEM and point-ID EDX characterization was done using an FEI NovaNanoSEM 450 with an accelerating voltage of 5 kV for the DE powders, and 15 kV for the C-coated nanoSi structures. All TEM/STEM analysis (and corresponding EDX mapping) in Fig. 3 was conducted at 300 kV accelerating voltage using an FEI Titan Themis system, equipped with an FEI SuperX energy dispersive spectrometer. TEM in Fig. 4 was conducted using an FEI Tecnai12 system. Powder XRD analysis was carried out using a PANalytical Empyrean with Cu -Kα standard radiation.

## Additional Information

**How to cite this article**: Campbell, B. *et al*. Carbon-Coated, Diatomite-Derived Nanosilicon as a High Rate Capable Li-ion Battery Anode. *Sci. Rep.* **6**, 33050; doi: 10.1038/srep33050 (2016).

## Figures and Tables

**Figure 1:**
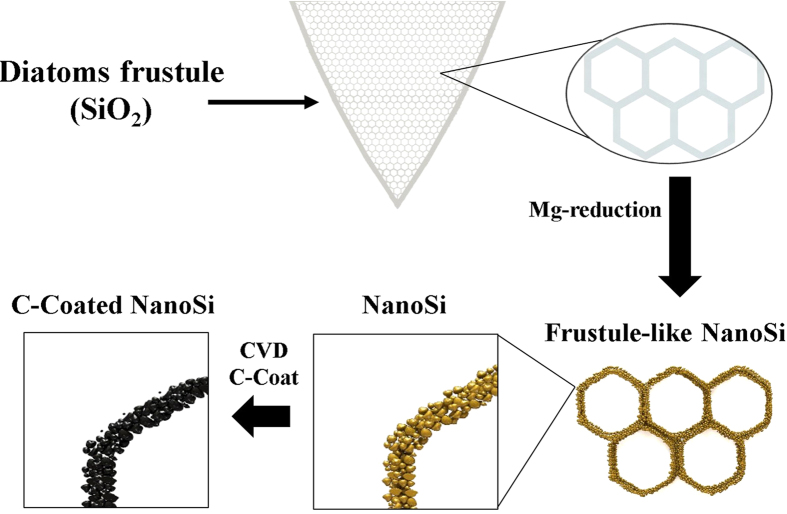
Schematic illustration of the process of obtaining C-coated, DE-derived, frustule-like nanoSi structures for use as Li-ion anode active material. Lauro Zavala is credited for the contribution of this artwork.
